# Effects of Secondary Metabolites from Pea on *Fusarium* Growth and Mycotoxin Biosynthesis

**DOI:** 10.3390/jof7121004

**Published:** 2021-11-24

**Authors:** Lakshmipriya Perincherry, Natalia Witaszak, Monika Urbaniak, Agnieszka Waśkiewicz, Łukasz Stępień

**Affiliations:** 1 Department of Plant-Pathogen Interaction, Institute of Plant Genetics, Polish Academy of Sciences, 60-479 Poznań, Poland; nwit@igr.poznan.pl (N.W.); murb@igr.poznan.pl (M.U.); lste@igr.poznan.pl (Ł.S.); 2 Department of Chemistry, Poznań University of Life Sciences, 60-625 Poznań, Poland; agnieszka.waskiewicz@up.poznan.pl

**Keywords:** *Fusarium*, mycotoxins, secondary metabolites

## Abstract

*Fusarium* species present ubiquitously in the environment are capable of infecting a wide range of plant species. They produce several mycotoxins targeted to weaken the host plant. While infecting some resistant plants, the host can alter the expression of toxin-related genes and accumulate no/very low amounts of mycotoxins. The ability of the host plant to modulate the biosynthesis of these toxins is entirely depending on the secondary metabolites produced by the plant, often as a part of systemic acquired resistance (SAR). A major role plays in the family of metabolites called phenyl propanoids, consisting of thousands of natural products, synthesized from the phenylalanine or tyrosine amino acids through a cascade of enzymatic reactions. They are also famous for inhibiting or limiting infection through their antioxidant characteristics. The current study was aimed at identifying the differentially expressed secondary metabolites in resistant (Sokolik) and susceptible (Santana) cultivars of pea (*Pisum sativum* L.) and understanding their roles in the growth and mycotoxin biosynthesis of two different *Fusarium* species. Although metabolites such as coumarin, spermidine, *p*-coumaric acid, isoorientin, and quercetin reduced the growth of the pathogen, a higher level of *p*-coumaric acid was found to enhance the growth of *F. proliferatum* strain PEA1. It was also noticeable that the growth of the pathogen did not depend on their ability to produce mycotoxins, as all the metabolites were able to highly inhibit the biosynthesis of fumonisin B_1_ and beauvericin.

## 1. Introduction

*Fusarium* species are soil-borne pathogens that rapidly build up in the soil and are persistent for many years [1]. They are considered as ubiquitous root-infecting hemibiotrophic pathogens that cause wilt diseases in several plant species such as cereals, legumes, vegetable and fruit crops. The wilt disease in pea is primarily caused by *Fusarium oxysporum* f.sp. pisi Schlecht (Van Hall) Snyd, reported in every country where the pea is cultivated [2]. Whenever the pathogen encounters a susceptible plant type under favorable conditions and optimum fungal load, the infection starts leading to substantial crop loss and yield reduction [3]. The infection process involves the pathogen penetrating the roots, entering the vascular system, and disrupting the water and nutrient transport. The infected host plant often shows orange to dark discoloration in the vascular tissues of the plant’s lower parts, whereas the above-ground symptoms include yellowing of leaves that curl downwards and wilt, especially during flowering or early pod formation stages. Infection at the early seedling stages kills the plant and in the later stages results in poor yield [4]. During the infection, the pathogen produces phytotoxic compounds such as mycotoxins, that weaken the host defense mechanisms, leading to root cell collapsing and venal chlorosis. It is also proposed that fusaric acid, a mycotoxin produced by *Fusarium*, is capable of reprograming the host’s metabolic pathways inducing senescence to enhance the severity of infection [5]. Specific effectors from the pathogen elicit host systemic acquired resistance (SAR), which involves the activation of salicylic acid (SA), jasmonic acid (JA), and ethylene (ET) signaling pathways. Generally, SA signaling is triggered when the host plant gets infected with biotrophic and hemibiotrophic pathogens [6]. Secondary metabolites primarily involved in disease resistance are phenylpropanoids and polyamines. Phenolic compounds including coumaric acid, coumarin, naringenin, cinnamic acid, quercetin, etc., are the largest group of plant antioxidant phytochemicals, from which a myriad of natural products are synthesized [7]. One such group called flavonoids helps plants to fight the pathogen through various mechanisms. They help to tighten the plant tissues by modulating auxin (IAA) activity, induce lignification and callose deposition, and are thought to chelate metals required by the pathogen’s enzymes, rendering them inactive [8]. Flavonoids along with polyamines suppress reactive oxygen species (ROS) produced during the infection. With an increase in the polyamine level, the H_2_O_2_ content in the cell is also increased and that resists the pathogen spread [9]. It has been identified that during infection, the pea plant produces several metabolites that are of low molecular weight, non-peptidyl molecules that may have a major role in disease resistance and tolerance. The major metabolites include polyamines (spermine, spermidine), γ-aminobutyric acid (GABA), modified jasmonates, alkaloids (trigonelline, trigonelline-hydrochloride). They also produce pisatin–a phytoalexin that is not present in healthy plants, but is synthesized in response to the infection and its activity is restricted to the colonized tissue or the neighboring cells [10].

Several plant secondary metabolites were found to have growth inhibitory effects in many plant-pathogenic fungi [11,12,13]. In contrast, some concentrations of plant phenolic compounds can stimulate the *Fusarium* growth [14]. Additionally, flavonoids were proven to have an inhibitory effect on the mycotoxins such as trichothecenes by inhibiting cytochrome P450 monooxygenase which catalyzes the conversion of trichodiene to oxygenated trichothecenes [15]. Phenolic compounds such as ferulic acid, p-hydroxybenzoic acid, and vanillic acid are proven to reduce the aurofusarin and zearalenone toxin levels in *Fusarium* species [14]. The main purpose of our research was to monitor the secondary metabolites produced in fungi-resistant (Sokolik) and susceptible (Santana) pea cultivars and how these metabolites alter the growth and mycotoxin biosynthesis in *Fusarium ozysporum* and *F. proliferatum* species.

## 2. Materials and Methods

### 2.1. Fungal Strains and Culture Conditions

The fungal pathogens *F. proliferatum* strain PEA1, PEA2 and *F. oxysporum* strain 34 OX and 1757 OX originally isolated from infected pea were grown on potato dextrose agar and incubated at 26 °C for 4–6 days. The strains were identified based on sequence analysis using ITS (Internal Transcribed Spacer) and TEF (Translation Elongation Factor 1α) primers in a previous study [16].

### 2.2. Infection Studies on Pea Seedlings

Seeds from both cultivars Sokolik and Santana were surface sterilized using 15% (*v*/*v*) of bleach (Sodium hypochlorite) in sterile distilled water for 30 s and then washed in sterile distilled water thrice to remove the bleach completely. Then the seeds were kept on sterile wet tissue inside a petri dish and stored in a dark chamber for three days to enhance germination. Once the seeds were germinated, the plates were transferred to a well-lit place and kept for four more days. On day seven, the seedlings were infected with five days old fungal mycelia grown on PDA cut out with a cork borer of diameter 5.5 mm. The seedlings were observed every day for the disease symptoms.

### 2.3. Metabolite Profiling of Pea Cultivars

#### 2.3.1. Sample Preparation

Sterilized seeds from Sokolik and Santana were germinated and the seedlings were transferred to sterilized soil. The plants were cultivated inside a growth chamber at 23 °C with a 16-h photoperiod. The plants were watered as required and were fertilized with a micro-macro nutrient solution consisting Ca(NO_3_)_2_ (4.18 g L^−1^); KNO_3_ (1.03 g L^−1^); KH_2_PO_4_ (0.35 g L^−1^); K_2_SO_4_ (0.43 g L^−1^); Mg(NO_3_)_2_ (0.51 g L^−1^); MgSO_4_ (0.63 g L^−1^), Fe chelate (0.5 g L^−1^) once after 15 days of planting. After one month, the whole plant was collected and were homogenized in liquid nitrogen. The homogenized samples were centrifuged at 10,000× *g* at 4 °C and the supernatant was collected. The extracts were filtered using 0.20 μm syringe filters (Chromafil PET20/15 MS, Macherey-Nagel, Dueren, Germany). Quality control samples (QC) were prepared by pooling the same amount of pea extracts.

#### 2.3.2. LC-MS Analysis

The analysis was performed using Acquity UPLC system (Waters Corporation, Milford, MA, USA) combined with Q-Exactive high-resolution mass spectrometer (Thermo Fisher, Waltham, MA, USA) with Orbitrap mass analyzer. Samples were maintained at 5 °C in autosampler and 7 μL of each was injected onto an ACQUITY UPLC BEH Shield RP18 column (150 × 2.1 mm, particle size 1.7 μm,) (Waters, Manchester, MA, USA) for chromatographic separation, with a flow rate of 0.35 μL min^−1^ at 50 °C. The mobile phases consisted of 0.1% (*v*/*v*) formic acid in water (eluent A) (LC-MS grade, Merck, Darmstadt, Germany) and acetonitrile (eluent B) (LC-MS grade, Merck). A multistep linear gradient was set as follows: 1% B–initial, 2% B–1.5 min, 65% B–10.5 min, 98% B–11.5 min, 98% B–12.5 min, and equilibration at 1% B for 2.5 min.

Mass spectrometry analysis was performed using heated electrospray ionization (HESI) in positive and negative modes. A 3.5 kV and 2.5 kV ion spray voltage was applied for positive and negative ionization, respectively. Ion source temperature was set to 320 °C in both ionization modes. Data were acquired in Full MS data-dependent MS^2^ mode in the range 100–1500 *m*/*z*. Full MS mode was operated at a resolution of 70,000 and ddMS^2^ mode at 17,500. Normalized collision energy in the ddMS^2^ experiment was set to 30%. According to the manufacturer’s guideline, the system was calibrated using standard solutions (ThermoFisher Scientific, Waltham, MA, USA) before analysis. Analysis was performed in three biological and two analytical replicates. Xcalibur software (ThermoFisher Scientific) was utilized for system operation, data acquisition as well as data analysis.

#### 2.3.3. Data Analysis

Raw data files (.RAW) were converted to abf format using *Reifycs* Abf Converter (https://www.reifycs.com/AbfConverter/, accessed on 11 March 2021). Data deconvolution, peak detection, and LOESS normalization based on QC samples were performed with the use of MSDial (version 4.00) [17]. Tentative metabolite identification was carried out based on accurate mass and ion fragmentation patterns in Xcalibur Qual Browser (ThermoFisher Scientific). Statistical analysis was conducted using Perseus (version 1.6.1.3.) [18]. Obtained MS-data was analyzed with T-test followed by false discovery rate (FDR) correction. Principal component analysis (PCA) as well as hierarchical cluster analysis on the basis of Euclidean distance calculation were used for data visualization.

### 2.4. Antifungal Activity Assays Using Pea Derived Metabolites

In vitro culture was prepared using 20 mL of potato dextrose agar (PDA) (Sigma Aldrich) inoculated with five-day-old fungal mycelia grown on PDA cut out with a cork borer of diameter 5.5 mm. Based on the results from metabolite profiling and availability of chemical standards, seven compounds including flavonoids and polyamines were selected. The metabolites dissolved in respective solvents were added to the PDA plates to make the final concentrations of 1 ng/mL, 10 ng/mL and 100 ng/mL, respectively. The plates were incubated at 26 °C in darkness for seven days. Direct measurement of fungal growth was carried out by measuring the fungal colony diameter every two days.

Based on the result from the plate assay, the metabolites and their corresponding concentrations were selected that either inhibited or promoted the growth of the fungus. The selected concentrations of metabolites (Table 1) were added to 50 mL of Czapek-Dox broth and inoculated with two plugs of 5-day-old fungal mycelia grown on PDA cut out with a cork borer of diameter 5.5 mm. A control was also kept without the addition of any metabolites. All the treatments were carried out in duplicates. The cultures were incubated at 26 °C in darkness for 14 days. Treatment with *p*-coumaric acid was carried out separately with 1 ng/mL and 100 ng/mL concentrations along with separate controls since it is one of the key metabolites in the phenyl propanoid pathway. Additionally, the results from *p*-coumaric acid treatments were compared to the separate controls, unlike all other treatments. On the last day, the culture supernatant was collected by centrifuging and they were stored at −80 °C for the mycotoxin analysis. Additionally, the total mycelia were collected frozen in liquid nitrogen and freeze-dried immediately using a lyophilizer until the water is completely removed from the samples. The weight of each sample was measured after the complete lyophilization.

### 2.5. Statistical Analysis

The statistical analyses were made with the Origin Pro 2020 program (Origin Lab Corporation, Northampton MA, USA). The data obtained from fungal growth and mycotoxin studies were analyzed with a one-way ANOVA with two replicates. Dunnett’s test was used to determine the significant differences between the treatments and the control, with *p* value < 0.05.

### 2.6. Mycotoxin Analysis

Mycotoxin standards (fumonisins B_1–3_ (FB_1–3_) and beauvericin (BEA)), water, and organic solvents of high purify for chromatographic analysis were purchased from Sigma-Aldrich (Steinheim, Germany). Before chromatographic analysis plant extracts were filtered through a 0.22 µm membrane (Chromafil PET 20/15/MS, Macherey-Nagel, Germany) and transferred to chromatography vials. Mycotoxins concentration was analyzed for each variant of plant extracts using a UPLC™ system (Acquity, Waters, Milford, MA, USA) connected with a triple quadrupole mass spectrometer (TQD; Waters Micromass, Manchester, UK) according to the methods described in detail earlier [19,20]. The limit of detection was 0.1 and 1.0 ng/µL for fumonisins and beauvericin, respectively. The qualitative and quantitative analysis of mycotoxins was performed in three analytical replicates. The results are expressed in percentage reduction in mycotoxins and were calculated using the equation below.

Mycotoxin reduction (%) = ((control − treatment)/control) × 100

## 3. Results

### 3.1. Infection Studies on Pea Seedlings

**Figure 1:**
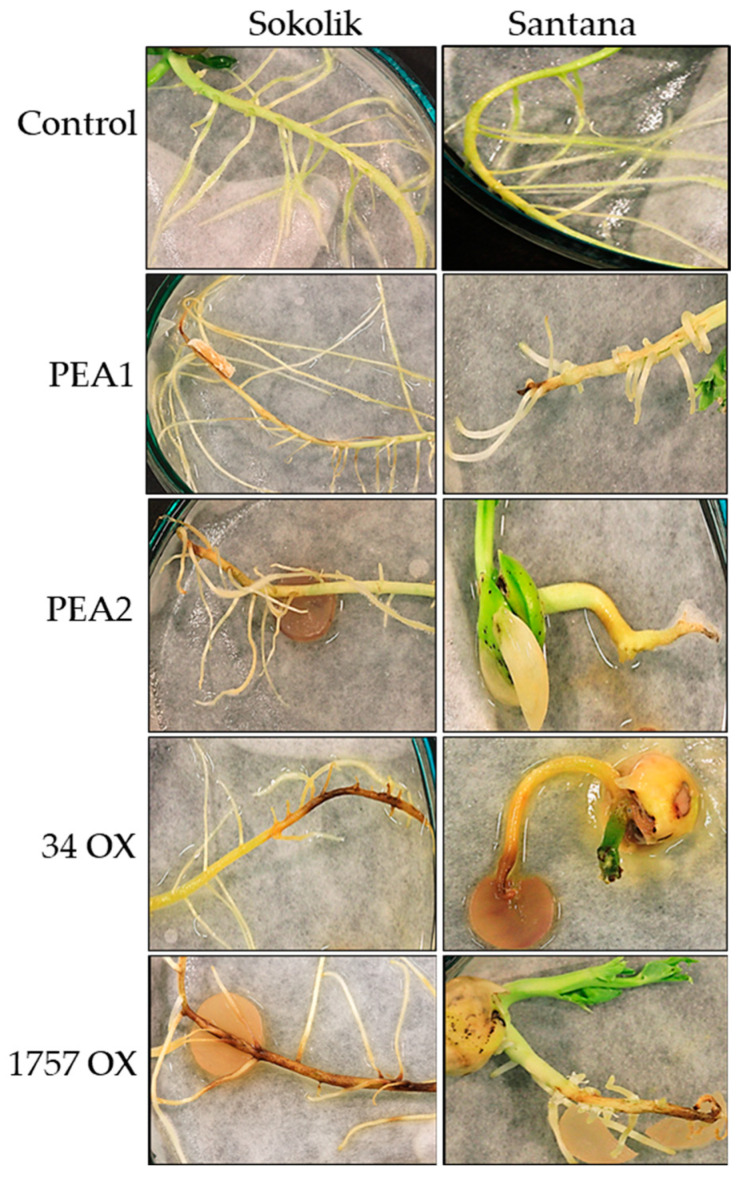
Roots of control plants and seedlings infected with *F. proliferatum* (PEA1, PEA2) and *F. oxysporum* (34 OX, 1757 OX) from Sokolik and Santana after 7 days of infection.

The results from seedling infection studies revealed that *F. oxysporum* is more pathogenic to pea than *F. proliferatum*. Figure 1 shows the pictures of control and infected seedlings from Sokolik and Santana after seven days of infection. The *F. proliferatum* strain PEA2 was more virulent than PEA1 and they could reduce the length and number of lateral roots of seedlings from susceptible cultivar Santana. The formation of lateral roots was partially inhibited in PEA2-infected seedlings and this strain was found to produce visible mycelium on the root tips. The *F. oxysporum* strains 34 OX and 1757 OX were highly pathogenic for both cultivars and can be considered as hypervirulent. The strain 34 OX could completely inhibit the growth of roots and within two days of infection, the pathogen destroyed the entire endosperm and the shoot.

### 3.2. Metabolite Profiling of Pea Cultivars

**Figure 2:**
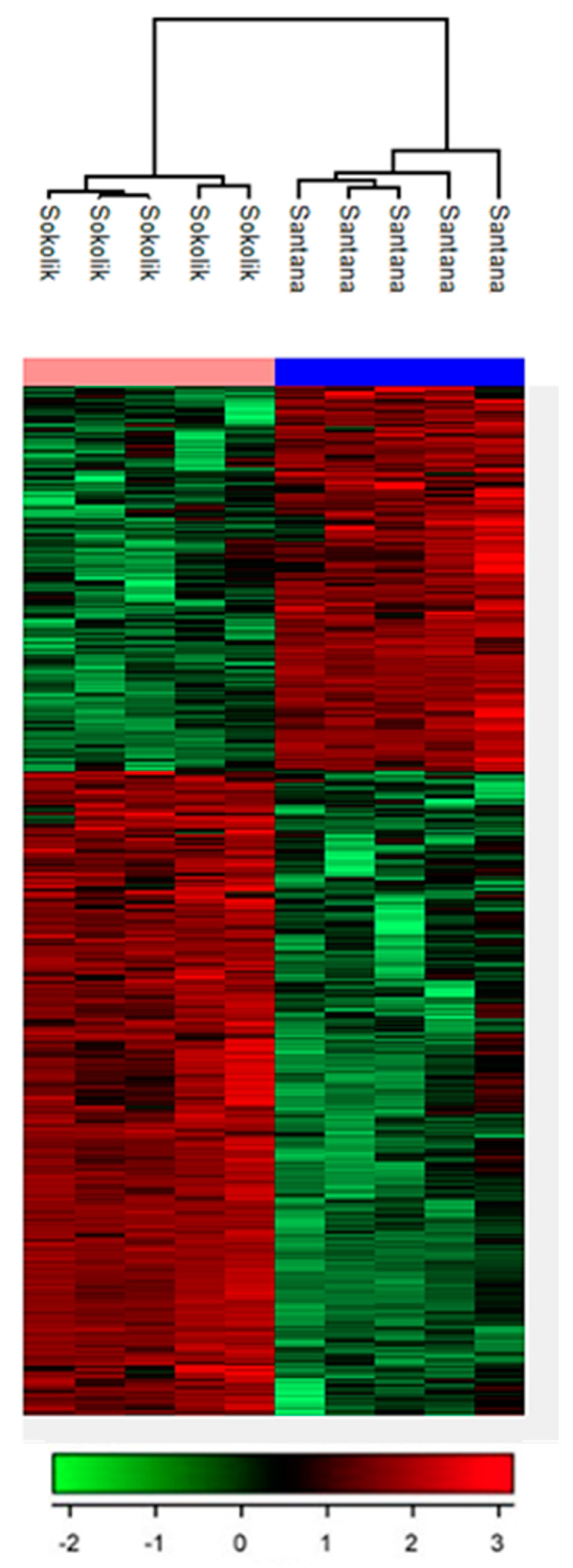
Heat map of significant metabolites reveals the metabolic signatures of Sokolik and Santana pea cultivars. The hierarchical clustering shows two distinct clusters of metabolites, which could discriminate between the two cultivars.

The LC-MS metabolomic analysis allowed for comparative analysis of metabolites present in the resistant and susceptible pea cultivars. (Figure 2). Based on assigning the mass to the results available in the databases connected to the program, it can be hypothesized that the differentiating metabolites in Sokolik are mostly flavonoids or products from the phenyl propanoid pathway that are involved in the systemic acquired resistance of plants against pathogens. Thereby, to carry out antifungal activity assays, a few most important compounds from the phenyl propanoid pathway and polyamine synthetic pathway were selected. The characteristics of metabolites found differentially in Sokolik and Santana are given in Table 2. Large number of metabolites made it impossible to include all IDs in the figure.

### 3.3. Antifungal Assay Using Pea-Derived Metabolites

**Figure 3:**
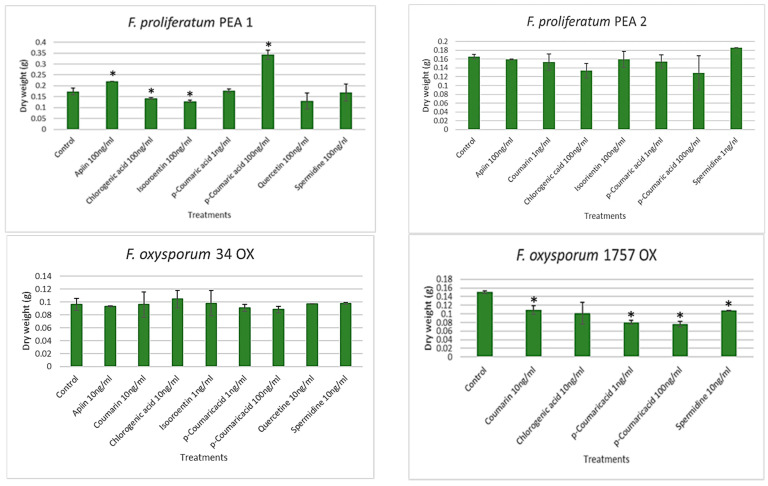
Dry mycelial weight (in grams) of *F. proliferatum* (PEA1 and PEA2) and *F. oxysporum*(34 OX and 1757 OX) strains on 14th day of culturing with various metabolites. Error bars represent standard error. * Statistically significant (*p* < 0.05).

The mycelial growth of *F. proliferatum* PEA1 and *F. oxysporum* 1757 OX were found to be inhibited by most of the selected combinations of metabolites (Figure 3). In PEA1 cultures, chlorogenic acid (100 ng/mL), Isoorientin (1 ng/mL), and quercetin (100 ng/mL) were found to limit the fungal growth compared to the control. Chlorogenic acid was found to reduce the growth by 19% whereas, chlorogenic acid and quercetin reduced the growth by 26%. However, no effect in growth was found in those cultures supplemented with apiin (100 ng/mL), *p*-coumaric acid (1 ng/mL), and spermidine (100 ng/mL). It was quite surprising to find that higher amounts of *p*-coumaric acid (100 ng/mL) promote fungal growth up to twice the weight of mycelia. There were no significant differences between the results from treated vs. the control cultures of PEA2 and 34 OX. An increased inhibition of growth was observed in the cultures of 1757 OX. Coumarin (10 ng/mL) and spermidine (10 ng/mL) reduced the growth of the fungus by 28% compared to the control. Although chlorogenic acid reduced the growth by 33%, the results were highly variable. In the treatments with 1757 OX, *p*-coumaric acid was found to be most effective with an overall growth reduction of 50%.

### 3.4. Mycotoxin Analysis

**Figure 4:**
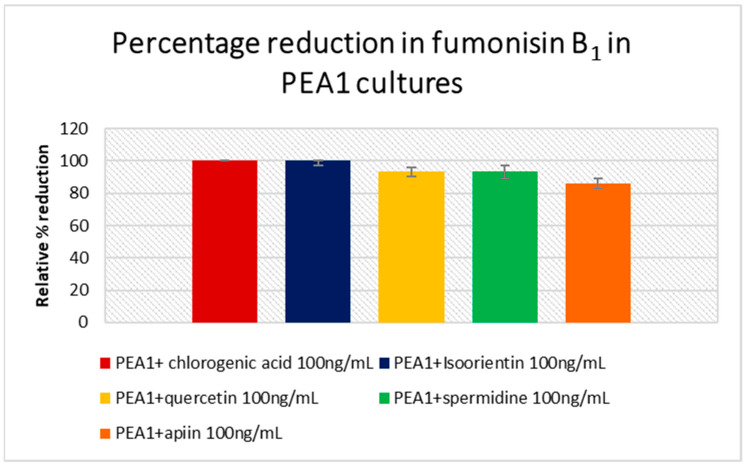
Percentage of fumonisin B_1_ reduction with respect to the control in *F. proliferatum* PEA1 cultures treated with various concentrations of host metabolites. Error bars represent standard error.

**Figure 5:**
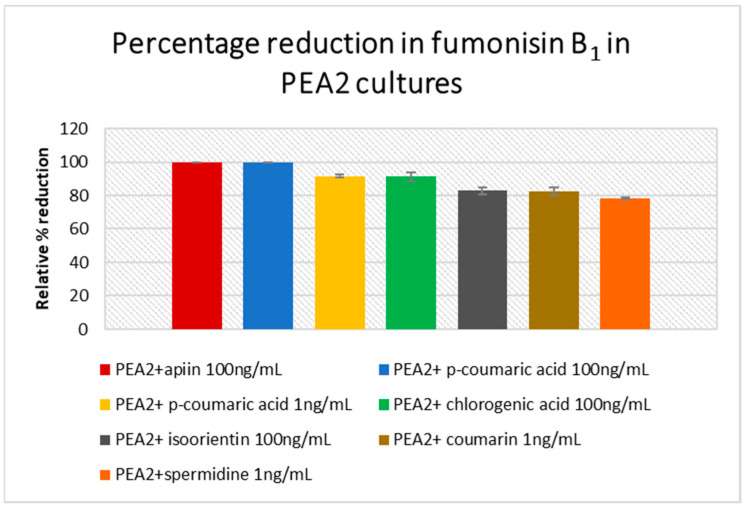
Percentage of fumonisin B_1_ reduction with respect to the control in *F. proliferatum* PEA2 cultures treated with various concentrations of host metabolites. Error bars represent standard error.

The amount of fumonisin B_1-3_ and beauvericin produced were analyzed in PEA1, PEA2, 34 OX and 1757 OX cultures. However, FB_2_ and FB_3_ were below the limit of detection. As one of the main producers of FB_1_, *F. proliferatum* cultures showed increased levels in their respective controls. PEA1 treated with 100 ng/mL of chlorogenic acid and isoorientin showed a 100% reduction in FB_1_ content compared to the control, whereas a high concentration of quercetin and spermidine could limit the production by 93% (Figure 4), (supplementary materials). While apiin (100 ng/mL) reduced FB_1_ levels by 86% in PEA1 cultures, a full inhibition was observed in PEA2 cultures (Figure 5). An 82% reduction in FB_1_ levels was also observed in PEA2 cultures supplemented with 100 ng/mL of isoorientin. Since treatment with *p*-coumaric acid was carried out separately with independent control, the levels of FB_1_ in PEA1 controls and in treatments were found to be zero and could not be included in the PEA1 graph (Figure 4). Even a low concentration (1 ng/mL) of spermidine and coumarin was just as effective as isoorientin to achieve an inhibition of around 78–82%. Out of all the metabolites studied, *p*-coumaric acid was found to be the best for reducing the fumonisin levels in PEA2 cultures, where both low and high concentrations could yield around 90–100% reduction compared to the control (supplementary materials). Similarly, chlorogenic acid (100 ng/mL) could also inhibit fumonisin production by 91%. Although 1757 OX did not produce fumonisins, 34 OX treated with apiin 10 ng/mL, isoorientin 1 ng/mL, and spermidine 10 ng/mL were found to produce FB_1_ in trace quantities and the results were statistically insignificant (*p* > 0.05) (supplementary materials). Beauvericin was found to be produced by control cultures of PEA1 and PEA2. Nevertheless, all the treatments showed a 100% inhibition in BEA levels. No cultures from *F. oxysporum* produced beauvericin under any treatments.

## 4. Discussion

*Fusarium* being a soil-inhabiting fungi are able to survive for more than 10 years as their spores are thick-walled and very hard. As mentioned earlier, they are capable of infecting a wide range of plant species including leguminous crops like pea. Although *F. oxysporum* is considered the cause of most wilt diseases [3,21], our study proves that *F. proliferatum* could also be a potential pathogen causing wilt disease in pea. The strains PEA1 and PEA2 could easily infect both the resistant and susceptible pea cultivars, but were not found to be as virulent as 34 OX and 1757 OX. The infection was observed to be initiated at the root tips gradually spreading to the upper root section. The most common entry points into the pea plant by the *Fusarium* pathogen were established to be the root tips, cotyledon nodes, or via wounded root openings [3].

It is well established that plant secondary metabolites could possibly reduce the growth and mycotoxin production in *Fusarium* species [13,14,22,23]. Hydroxylated polyphenolic compounds or flavonoids are widely distributed in the plant kingdom. They play a major role in plant defense mechanisms against bacterial and fungal pathogens [24]. These compounds are biosynthesized through the type III polyketide synthase pathway. The key metabolites involved in the pathway are phenylalanine, trans-cinnamic acid, *p*-coumaric acid, naringenin, quercetin, apigenin, etc. [13]. The metabolic profiling of Sokolik and Santana revealed that the increased resistance of Sokolik to the pathogen could be accredited to the high level of Phenylpropanoids. Hence, we selected the key metabolites involved in the phenylpropanoid pathway and a few of their derivatives for studying how they alter the pathogen’s growth and metabolism. Higher concentrations of hydroxy cinnamic acids such as chlorogenic acid account for resistance towards several plant pathogens such as *Phytophthora infestans, Streptomyces scabies and Verticillium alboatrum,* while their lower concentrations stimulate the growth of pathogens including *F. solani* [25]. In our studies, 100 ng/mL concentration of chlorogenic acid and isoorientin was found to inhibit the PEA1 growth effectively and a reduced concentration promotes the growth of 34 OX cultures. In spite of being a member in the same category, a higher concentration of *p*-coumaric acid could induce increased growth in PEA1 cultures. Flavonoids and allied phenolic compounds such as isoorientin, coumarins, lignans, and polyphenols are involved in the defense systems such as pathogen and UV resistance. These compounds are also famous for altering the fungal membrane permeability and interaction with membrane proteins leading to the subsequent disruption of membranes [26]. Another mechanism resulting from their antioxidant property is that flavonoids quench the reactive oxygen species (ROS) generated during the infection they also inhibit plant cell wall degrading enzymes produced by the pathogens. In the current study, none of the metabolites monitored inhibited the growth of PEA2 and 34 OX strains. PEA2 and 34 OX, being the virulent strain of *F. proliferatum* and *F. oxysporum*, respectively, were found to be unresponsive to the metabolites in terms of growth. However, it was noticed that the metabolites could alter the mycotoxin biosynthesis in all the strains including 34 OX and 1757 OX. Phenolics such as ellagic acid and isoeugenol were recently reported to inhibit growth and fumonisin B accumulation in *F. verticillioides* and were found to regulate the biosynthesis at the transcription level by altering the expression of *FUM* genes [27]. In our case, flavonoids and phenolic compounds such as isoorientin, chlorogenic acid and *p*-coumaric acid completely inhibited the FB_1_ production in *F. proliferatum* cultures. All other metabolites could efficiently inhibit fumonisin production up to 91%. Along with inhibiting fumonisin production, all the metabolites reduced the beauvericin levels by 100% compared to the control in *F. proliferatum*. Beauvericin belongs to the cyclic hexadepsipeptide group of mycotoxins produced by a number of *Fusarium* species [28,29] and is one of the toxic compound that disrupts the membrane structure of host plants, induce apoptosis and enhance the infection severity [30]. This is the first report on phenylpropanoids inhibiting beauvericin biosynthesis in *F. proliferatum*. The ability of phenyl propanoid compounds observed in resistant pea phenotypes in inhibiting growth and mycotoxin biosynthesis suggests further investigations to study the molecular basis of inhibition mechanisms. Such studies could pave the way for the development of effective new disease control treatments.

## Figures and Tables

**Table 1:** Selected concentrations of metabolites for the liquid culture studies (indicated by √) based on fungal growth on PDA.

	PEA1			PEA2			34 OX			1757 OX		
Conc ng/mL	1	10	100	1	10	100	1	10	100	1	10	100
Apiin			√			√		√				
Coumarin				√				√			√	
Chlorogenic acid			√			√		√			√	
Isoorientin			√			√	√					
*p*-coumaric acid	√		√	√		√	√		√	√		√
Quercetin			√					√				
Spermidine			√	√				√			√	

**Table 2:** Identification characteristics of the selected metabolites found differentially present in the extracts of Santana and Sokolik. (red color denotes higher quantities of the metabolite, green color denotes lower quantities of the metabolite).

RT [min]	Metabolite Name	Ionization	Molecular Formula	Mass Calculated	Mass Measured	Δ [ppm]	Fragment Ions	PubChem ID	Santana	Sokolik
5.87	Apiin (Apigenin 7-O-diglucoside)	[M−H]^−^	C_26_H_28_O_14_	563.1406	563.1412	1.0774	473, 563, 284	280746		
7.05	Coumarin	[M+H]^+^	C_9_H_6_O_2_	147.044	147.0441	−0.5453	103	323		
5.05	Chlorogenic Acid	[M−H]^−^	C_16_H_18_O_9_	353.0878	353.0878	−0.2770	191	1794427		
6.59	Isoorientin	[M+H]^+^	C_21_H_20_O_11_	449.1078	449.1075	−0.6544	449, 287	114776		
3.67	*p*-coumaric Acid	[M+H]^+^	C_9_H_8_O_3_	165.0545	165.0546	−0.5070	147	637542		
6.28	Quercetin	[M+H]^+^	C_15_H_10_O_7_	303.0499	303.0499	−0.2092	229, 153	5280343		
5.08	Spermidine	[M+H]^+^	C_7_H_19_N_3_	146.1652	146.1651	−0.5070	129, 112, 172	1102		

## Data Availability

The data presented in this study are available in the article and Supplementary files.

## Supplementary Materials

The following are available online at https://www.mdpi.com/article/10.3390/jof7121004/s1, Tables S1–S9: Effect of isoorientin (S1), chlorogenic acid (S2), apiin (S3), quercetin (S4), coumarin (S5), spermidine (S6), *p*-coumaric acid (S7) in growth of *F. proliferatum* and *F. oxysporum,* FB1 and beauvericin produced by *F. proliferatum* and *F. oxysporum* after the addition of metabolites (S8) and after addition of *p*-coumaric acid (S9).
