# Supplementary material for: Effects of Secondary Metabolites from Pea on Fusarium Growth and Mycotoxin Biosynthesis
Source: J Fungi (Basel). 2021 Nov 24;7(12):1004. doi: 10.3390/jof7121004 (PMC8706721; doi:10.3390/jof7121004)
Supplement: Supplementary file 1 [file jof-07-01004-s001.zip › jof-1443427-supplementary.pdf]

Effects of Secondary Metabolites from Pea on *Fusarium* Growth and Mycotoxin Synthesis

Table S1: Effect of isoorientin (1ng/ml, 10ng/ml and 100ng/ml) in the growth of *F. proliferatum* (PEA1 and PEA2) and *F. oxysporum* (34 OX and 1757 OX).

Isoorientin	PEA1				PEA2				34 OX				1757 OX			
**Day 2**	Control	1ng/ml	10ng/ml	100ng/ml	Control	1ng/ml	10ng/ml	100ng/ml	Control	1ng/ml	10ng/ml	100ng/ml	Control	1ng/ml	10ng/ml	100ng/ml
Mean (cm)	1.7	1.65	1.7	1.85	1.75	1.9	1.8	2	1.6	1.45	1.25	1.3	2.1	2	2.1	2.2
SD	0	0.07	0	0.07	0.07	0	0	0	0	0.07	0.07	0	0	0	0	0.14
**Day 4**	Control	1ng/ml	10ng/ml	100ng/ml	Control	1ng/ml	10ng/ml	100ng/ml	Control	1ng/ml	10ng/ml	100ng/ml	Control	1ng/ml	10ng/ml	100ng/ml
Mean (cm)	3.7	3.8	3.8	3.95	3.8	4	3.95	4.05	3.45	3.2	3.05	3.15	4.8	4.7	4.75	4.8
SD	0.14	0	0	0.07	0	0.14	0.07	0.07	0.07	0	0.07	0.07	0	0	0.07	0
**Day 6**	Control	1ng/ml	10ng/ml	100ng/ml	Control	1ng/ml	10ng/ml	100ng/ml	Control	1ng/ml	10ng/ml	100ng/ml	Control	1ng/ml	10ng/ml	100ng/ml
Mean (cm)	5.35	5.8	5.8	6	5.6	5.9	5.7	5.9	5.3	4.55	4.7	4.7	6.95	6.8	6.95	7
SD	0.2	0	0	0	0	0	0	0	0	0.07	0	0	0.07	0	0.07	0

Table S2: Effect of chlorogenic acid (1ng/ml, 10ng/ml and 100ng/ml) in the growth of F. proliferatum (PEA1 and PEA2) and F. oxysporum (34 OX and 1757 OX).

Chlorogenic acid	PEA1				PEA2				34 OX				1757 OX			
**Day 2**	Control	1ng/ml	10ng/ml	100ng/ml	Control	1ng/ml	10ng/ml	100ng/ml	Control	1ng/ml	10ng/ml	100ng/ml	Control	1ng/ml	10ng/ml	100ng/ml
Mean (cm)	1.7	1.7	1.9	1.65	1.75	2.15	1.8	1.9	1.6	1.4	1.35	1.35	2.1	2.2	2.2	2.1
SD	0	0	0	0.07	0.07	0.63	0	0	0	0	0.07	0.07	0	0.14	0	0
**Day 4**	Control	1ng/ml	10ng/ml	100ng/ml	Control	1ng/ml	10ng/ml	100ng/ml	Control	1ng/ml	10ng/ml	100ng/ml	Control	1ng/ml	10ng/ml	100ng/ml
Mean (cm)	3.7	3.85	4	3.6	3.8	3.6	3.8	4	3.45	3.15	3.15	3.25	4.8	4.75	4.75	4.65
SD	0.14	0.07	0	0	0	0	0	0.14	0.07	0.07	0.07	0.07	0	0.07	0.07	0.07
**Day 6**	Control	1ng/ml	10ng/ml	100ng/ml	Control	1ng/ml	10ng/ml	100ng/ml	Control	1ng/ml	10ng/ml	100ng/ml	Control	1ng/ml	10ng/ml	100ng/ml
Mean (cm)	5.35	5.8	5.8	5.9	5.6	5.6	5.7	5.8	5.3	4.75	4.5	4.8	6.95	6.9	6.8	6.7
SD	0.21	0	0	0	0	0	0	0.14	0	0.07	0	0	0.07	0	0	0

Table S3: Effect of apiin (1ng/ml, 10ng/ml and 100ng/ml) in the growth of F. proliferatum (PEA1 and PEA2) and F. oxysporum (34 OX and 1757 OX).

Apiin	PEA1				PEA2				34 OX				1757 OX			
**Day 2**	Control	1ng/ml	10ng/ml	100ng/ml	Control	1ng/ml	10ng/ml	100ng/ml	Control	1ng/ml	10ng/ml	100ng/ml	Control	1ng/ml	10ng/ml	100ng/ml
Mean (cm)	1.7	1.7	1.65	1.55	1.75	1.7	1.6	1.55	1.6	1.45	1.3	1.1	2.1	2.2	2.3	2.15
SD	0	0	0.21	0.07	0.07	0	0	0.07	0	0.07	0.14	0	0	0	0	0.07
**Day 4**	Control	1ng/ml	10ng/ml	100ng/ml	Control	1ng/ml	10ng/ml	100ng/ml	Control	1ng/ml	10ng/ml	100ng/ml	Control	1ng/ml	10ng/ml	100ng/ml
Mean (cm)	3.8	3.65	3.8	3.8	3.8	3.8	3.8	3.55	3.45	3.3	3.2	3	4.8	4.9	4.8	4.8
SD	0	0.07	0	0	0	0	0	0.07	0.07	0	0	0	0	0	0	0
**Day 6**	Control	1ng/ml	10ng/ml	100ng/ml	Control	1ng/ml	10ng/ml	100ng/ml	Control	1ng/ml	10ng/ml	100ng/ml	Control	1ng/ml	10ng/ml	100ng/ml
Mean (cm)	5.35	5.6	5.7	5.7	5.6	5.5	5.5	5.3	5.3	4.7	4.7	4.7	6.95	7	7	7
SD	0.21	0.14	0	0	0	0	0.14	0.14	0	0	0	0	0.07	0	0	0

Table S4: Effect of quercetin (1ng/ml, 10ng/ml and 100ng/ml) in the growth of F. proliferatum (PEA1 and PEA2) and F. oxysporum (34 OX and 1757 OX).

Quercetin	PEA1				PEA2				34 OX				1757 OX			
**Day 2**	Control	1ng/ml	10ng/ml	100ng/ml	Control	1ng/ml	10ng/ml	100ng/ml	Control	1ng/ml	10ng/ml	100ng/ml	Control	1ng/ml	10ng/ml	100ng/ml
Mean (cm)	1.7	1.65	1.6	1.55	1.75	1.8	1.8	1.9	1.6	1.3	1.3	1.35	2.1	2.35	2.1	2.2
SD	0	0.07	0	0.07	0.07	0	0	0	0	0	0	0.07	0	0.07	0	0.14
**Day 4**	Control	1ng/ml	10ng/ml	100ng/ml	Control	1ng/ml	10ng/ml	100ng/ml	Control	1ng/ml	10ng/ml	100ng/ml	Control	1ng/ml	10ng/ml	100ng/ml
Mean (cm)	3.7	3.45	3.6	3.8	3.8	3.8	3.9	3.8	3.45	3.35	3.5	3.4	4.8	4.8	4.6	4.65
SD	0.14	0.21	0	0	0	0	0.14	0	0.07	0.07	0	0	0	0	0.14	0.07
**Day 6**	Control	1ng/ml	10ng/ml	100ng/ml	Control	1ng/ml	10ng/ml	100ng/ml	Control	1ng/ml	10ng/ml	100ng/ml	Control	1ng/ml	10ng/ml	100ng/ml
Mean (cm)	5.35	5.5	5.55	5.6	5.6	5.6	5.7	5.6	5.3	5	5.35	5	6.95	6.95	6.9	6.9
SD	0.21	0	0.07	0	0	0	0	0.14	0	0	0.21	0	0.07	0.07	0	0

Table S5: Effect of coumarin (1ng/ml, 10ng/ml and 100ng/ml) in the growth of F. proliferatum (PEA1 and PEA2) and F. oxysporum (34 OX and 1757 OX).

Coumarin	PEA1				PEA2				34 OX				1757 OX			
**Day 2**	Control	1ng/ml	10ng/ml	100ng/ml	Control	1ng/ml	10ng/ml	100ng/ml	Control	1ng/ml	10ng/ml	100ng/ml	Control	1ng/ml	10ng/ml	100ng/ml
Mean (cm)	1.7	1.5	1.55	1.55	1.75	2.1	1.7	1.6	1.6	1.5	1.4	1.35	2.1	2.35	2	2.35
SD	0	0	0.07	0.07	0.07	0	0	0	0	0	0	0.07	0	0.07	0	0.07
**Day 4**	Control	1ng/ml	10ng/ml	100ng/ml	Control	1ng/ml	10ng/ml	100ng/ml	Control	1ng/ml	10ng/ml	100ng/ml	Control	1ng/ml	10ng/ml	100ng/ml
Mean (cm)	3.7	3.7	3.85	3.6	3.8	4.2	3.85	3.6	3.45	3.35	3.1	3.05	4.8	4.9	4.7	4.7
SD	0.14	0	0.07	0	0	0	0.07	0	0.07	0.07	0	0.07	0	0	0	0
**Day 6**	Control	1ng/ml	10ng/ml	100ng/ml	Control	1ng/ml	10ng/ml	100ng/ml	Control	1ng/ml	10ng/ml	100ng/ml	Control	1ng/ml	10ng/ml	100ng/ml
Mean (cm)	5.35	5.55	5.3	5.3	5.6	6	5.7	5.65	5.3	5.05	4.8	4.8	6.95	7	6.6	6.75
SD	0.21	0.07	0	0	0	0	0	0.07	0	0.07	0	0	0.07	0	0	0.07

Table S6: Effect of spermidine (1ng/ml, 10ng/ml and 100ng/ml) in the growth of F. proliferatum (PEA1 and PEA2) and F. oxysporum (34 OX and 1757 OX).

spermidine	PEA1				PEA2				34 OX				1757 OX			
**Day 2**	Control	1ng/ml	10ng/ml	100ng/ml	Control	1ng/ml	10ng/ml	100ng/ml	Control	1ng/ml	10ng/ml	100ng/ml	Control	1ng/ml	10ng/ml	100ng/ml
Mean (cm)	1.7	1.8	1.5	1.5	1.75	2	1.6	1.8	1.6	1.5	1.35	1.25	2.1	2.45	2.35	2.3
SD	0	0	0	0	0.07	0	0	0.14	0	0	0.07	0.07	0	0.07	0.07	0
**Day 4**	Control	1ng/ml	10ng/ml	100ng/ml	Control	1ng/ml	10ng/ml	100ng/ml	Control	1ng/ml	10ng/ml	100ng/ml	Control	1ng/ml	10ng/ml	100ng/ml
Mean (cm)	3.7	4.1	3.7	3.9	3.8	4.3	3.8	3.9	3.45	3.55	3.25	3.3	4.8	5.1	5	4.95
SD	0.14	0.14	0.14	0	0	0	0	0.14	0.07	0.07	0.07	0	0	0	0	0.07
**Day 6**	Control	1ng/ml	10ng/ml	100ng/ml	Control	1ng/ml	10ng/ml	100ng/ml	Control	1ng/ml	10ng/ml	100ng/ml	Control	1ng/ml	10ng/ml	100ng/ml
Mean (cm)	5.35	5.85	5.5	5.85	5.6	6.1	5.5	5.65	5.3	5.1	4.7	4.7	6.95	7.45	7	6.9
SD	0.21	0.07	0.14	0.07	0	0	0	0.21	0	0	0	0	0.07	0.07	0	0.14

Table S7: Effect of p- coumaric acid (1ng/ml, 10ng/ml and 100ng/ml) in the growth of F. proliferatum (PEA1 and PEA2) and F. oxysporum (34 OX and 1757 OX).

p-Coumaric acid	PEA1				PEA2				34 OX				1757 OX			
**Day 2**	Control	1ng/ml	10ng/ml	100ng/ml	Control	1ng/ml	10ng/ml	100ng/ml	Control	1ng/ml	10ng/ml	100ng/ml	Control	1ng/ml	10ng/ml	100ng/ml
Mean (cm)	1.7	1.7	1.75	1.6	1.75	1.75	1.65	1.65	1.6	1.55	1.6	1.65	2.1	2.15	2.2	2.15
SD	0	0	0.07	0	0.07	0.07	0.07	0.07	0	0.07	0	0.07	0	0.07	0	0.07
**Day 4**	Control	1ng/ml	10ng/ml	100ng/ml	Control	1ng/ml	10ng/ml	100ng/ml	Control	1ng/ml	10ng/ml	100ng/ml	Control	1ng/ml	10ng/ml	100ng/ml
Mean (cm)	3.8	3.9	3.8	3.75	3.8	3.95	3.95	3.7	3.45	3.7	3.55	3.5	4.8	4.6	4.6	4.85
SD	0	0	0	0.07	0	0.07	0.07	0	0.07	0	0.07	0.28	0	0.14	0.14	0.07
**Day 6**	Control	1ng/ml	10ng/ml	100ng/ml	Control	1ng/ml	10ng/ml	100ng/ml	Control	1ng/ml	10ng/ml	100ng/ml	Control	1ng/ml	10ng/ml	100ng/ml
Mean (cm)	5.35	5.6	5.55	5.55	5.6	5.5	5.5	5.25	5.3	5.55	5.65	5.4	6.95	6.85	6.8	6.925
SD	0.21	0	0.07	0.07	0	0	0.14	0.07	0	0.07	0.07	0	0.07	0.07	0	0.03

Table S8: FB_1 and beauvericin produced in *F. proliferatum* (PEA1 and PEA2) *F. oxysporum* (34 OX and 1757 OX) liquid cultures upon addition of various concentrations of metabolites (mean values and standard errors). Calculated from replicate treatments, *statistically significant.

Treatments	Fumonisin B_1 (ng/μL)		Beauvericin (ng/μL)	
	Mean	SE	Mean	SE
PEA1 control	5.21	0.63	1.21	0.18
PEA1 + apiin 100 ng/mL	0.73 *	0.24	0 *	0
PEA1 + chlorogenic acid 100 ng/mL	0 *	0	0 *	0
PEA1 + isoorientin 100 ng/mL	0 *	0	0 *	0
PEA1 + quercetin 100 ng/mL	0.35 *	0.02	0 *	0
PEA1 + spermidine 100 ng/mL	0.35 *	0.177046	0 *	0
PEA2 control	2.58	0.46	1.07	0.54
PEA2 +apiin 100 ng/mL	0 *	0	0 *	0
PEA2 + coumarin 1 ng/mL	0.45 *	0.003	0 *	0
PEA2 + chlorogenic acid 100 ng/mL	0.23 *	0.02	0 *	0
PEA2 + isoorientin 100 ng/mL	0.45 *	0.02	0 *	0
PEA2 + spermidine 1 ng/mL	0.57 *	0.09	0 *	0
34 OX control	0	0	0	0
34 OX + apiin 10 ng/mL	0.58	0.29	0	0
34 OX + coumarin 10 ng/mL	0	0	0	0
34 OX + chlorogenic acid 10 ng/mL	0	0	0	0
34 OX + isoorientin 1 ng/mL	0.27	0.14	0	0
34 OX + quercetin 100 ng/mL	0	0	0	0
34 OX + spermidine 10 ng/mL	0.96	0.48	0	0
1757 OX control	0	0	0	0
1757 OX + coumarin 10 ng/mL	0	0	0	0
1757 OX + chlorogenic acid 100 ng/mL	0	0	0	0
1757 OX + spermidine 1 ng/mL	0	0	0	0

Table S9: FB_1 and beauvericin produced in *F. proliferatum* (PEA1 and PEA2) *F. oxysporum* (34 OX and 1757 OX) liquid cultures upon addition of various concentrations of p-coumaric acid (mean values and standard errors). Calculated from replicate treatments, *statistically significant.

Treatments	Fumonisin B_1 (ng/μL)		Beauvericin (ng/μL)	
	Mean	SE	Mean	SE
PEA1 control	0	0	0	0
PEA1 + p-coumaric acid 1ng/mL	0	0	0	0
PEA1 + p-coumaric acid 100 ng/mL	1.61	0.80	0	0
PEA2 control	50.73	4.91	1.24	0.62
PEA2 + p-coumaric acid 1 ng/mL	4.30 *	1.74	0 *	0
PEA2 + p-coumaric acid 100 ng/mL	0 *	0	0 *	0
34 OX control	0	0	0	0
34 OX + p-coumaric acid 1 ng/mL	0	0	0	0
34 OX + p-coumaric acid 100 ng/mL	0	0	0	0
1757 OX control	0	0	0	0
1757 OX + p-coumaric acid 1 ng/mL	0	0	0	0
1757 OX + p-coumaric acid 100 ng/mL	0	0	0	0
